# The regulatory role of m^6^A methylation modification in metabolic syndrome pathogenesis and progression

**DOI:** 10.3389/fphys.2024.1271874

**Published:** 2024-03-14

**Authors:** Diwen Ye, Yongjiao Zhang, Bingyang Zhang, Junjun Liu, Tianshu Wei, Sumei Lu

**Affiliations:** ^1^ Department of Laboratory Medicine, The First Affiliated Hospital of Shandong First Medical University, Jinan, China; ^2^ School of Medical Laboratory, Weifang Medical University, Weifang, Shandong, China

**Keywords:** m 6 A, nonalcoholic fatty liver disease, diabetes, atherosclerosis, inflammatory response, autophagy, programmed cell death, metabolic syndrome

## Abstract

Metabolic syndromes are characterized by various complications caused by disrupted glucose and lipid metabolism, which are major factors affecting the health of a population. However, existing diagnostic and treatment strategies have limitations, such as the lack of early diagnostic and therapeutic approaches, variability in patient responses to treatment, and cost-effectiveness. Therefore, developing alternative solutions for metabolic syndromes is crucial. N6-methyladenosine (m^6^A) is one of the most abundant modifications that determine the fate of RNA. m^6^A modifications are closely associated with metabolic syndrome development and present novel prospects for clinical applications. Aberrant m^6^A modifications have been detected during inflammatory infiltration, apoptosis, autophagy, iron sagging, necrosis, and scorching during metabolic syndrome pathogenesis and progression. However, few reviews have systematically described the correlation between m^6^A modifications and these factors concerning metabolic syndrome pathogenesis and progression. This study summarizes the m^6^A methylation regulators and their roles in metabolic syndrome development, highlighting the potential of m^6^A modification as a biomarker in metabolic disorders.

## Highlights

1. m^6^A RNA regulators are closely associated with metabolic syndrome development and present novel prospects for clinical applications.
2. m^6^A modification-induced inflammatory responses may potentially be valuable in metabolic syndromes.
3. Programmed cell death controlled by m^6^A regulates metabolic syndromes.

## 1 Introduction

Metabolic sydromes are a group of disorders caused by glucolipid metabolism dysregulation, with symptoms such as obesity, hyperglycemia, hyperlipidemia, and nonalcoholic fatty liver severely affecting physical health. Furthermore, these symptoms are interrelated metabolic risk factors directly contributing to atherosclerotic cardiovascular disease development and increasing the risk of type 2 diabetes mellitus (T2DM) and its complications (Wang et al., 2015; Shi et al., 2017). Recently, research into the pathogenic mechanisms of metabolic sydromes has increasingly focused on excessive lipid accumulation, inflammatory responses, autophagy, apoptosis and epigenetic modifications (Shi et al., 2017; Huang et al., 2018; Wu et al., 2020).

Several epigenetic modifications exist, including deoxyribonucleic acid (DNA) methylation, histone modifications, messenger RNA (mRNA), and noncoding RNA chemical modifications. Methylated RNA is reportedly functional in pathophysiological processes at all stages of life. Adenylate methylation modifications account for over half of the total methylated ribonucleotides in cellular RNA and 0.1%–0.4% of all adenosines (Goldberg et al., 2007). N6-methyladenosine (m^6^A), a widespread trans-epigenetic adenylate modification, has become relevant to the specific mechanisms of metabolic sydrome pathogenesis (Li et al., 2017; Wu et al., 2020). m^6^A modification is a dynamic and reversible biological progress owing to the interaction between “writers” and “erasers.” The “writers” mean methyltransferase, such as methyltransferase-like 3 (METTL3), methyltransferase-like 14 (METTL14). The “erasers” mean demethylase, such as fat mass and obesity-associated protein (FTO) (Jia et al., 2011) and ALKB homolog 5 (ALKBH5). Also, “readers” are the third critical factor which recognizes m^6^A modifications and ultimately paly function.

m^6^A primarily controls post-transcriptional gene expression and is involved in DNA repair, cellular reprogramming, cell differentiation, cellular stress responses, and programmed cell death (PCD) (Wu et al., 2020). In addition, m^6^A is highly conserved in the 3′untranslated region (UTR) and the consensus motif RRACH in the coding region (Desrosiers et al., 1974; Wu et al., 2020). m^6^A methylation regulates almost all aspects of mRNA metabolism through “readers,” including nuclear export (Roundtree et al., 2017a; Roundtree et al., 2017b), stability (Wang et al., 2015), translation (Huang et al., 2018), and pre-mRNA processing to mRNA decay (Li et al., 2017; Shi et al., 2017). As reported, m^6^A methylation has been a hot research field in metabolic sydromes. In high-fat diet-induced fatty liver in mice, m^6^A hyper-methylated genes were significantly enriched in processes and pathways associated with lipid metabolism, such as fatty acid synthesis, triglyceride metabolism, and the PPAR signaling pathways (Zhong et al., 2020). m^6^A methylation controls the insulin IGF1-AKT-PDX1 pathway and T2DM is exacerbated by targeting METTL3 or METTL14 to reduce m^6^A levels and decrease AKT phosphorylation and PDX1 protein levels (De Jesus et al., 2019). YTHDF2 accelerates JAK2 mRNA degradation and attenuates JAK2-STAT3-C/EBPβ signaling. In our previous studies, we found that m^6^A ultimately contributes to the development of NAFLD and insulin resistance by regulating the expression of CYP2B6 (Li et al., 2023). Existing literature indicated the potential value of m^6^A methylation as novel targets in metabolic sydromes treatment.

In the present study, we review the relationship between m^6^A and metabolic syndromes and summarize the role of RNA m^6^A modification in metabolic syndrome pathogenesis and progression. We first introduce the role of m^6^A methylation “writers,” “erasers,” and “readers,” then the association of m^6^A methylation modification with inflammatory response, PCD, and other essential biological functions in metabolic syndromes. For the literature review, we searched the PubMed database of the NCBI Homepage using the keywords “m^6^A and inflammation”, “m^6^A and autophagy”, “m^6^A and programmed cell death,” and “m^6^A and metabolic syndrome” to filter the key publications related to metabolic syndromes and categorize and extract the content of interest. This study can provide evidence for the clinical application of m^6^A methylation in preventing and treating metabolic syndromes.

## 2 m^6^A-related component modulation

**FIGURE 1:**
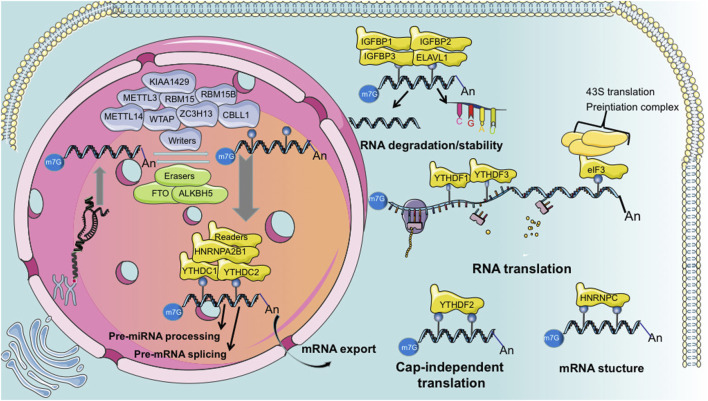
Summarized schematic of “writer,” “eraser,” and “reader” functions during m^6^A modification.

We summarized the components of m^6^A methylation process in Figure 1. The primary molecules involved in m^6^A are methyltransferases that methylate RNA, called the “writers”; demethylases that remove m^6^A, called the “erasers”; and “readers” which recognize m^6^A modifications and ultimately play functions. Thus, m^6^A modification is dynamically and reversibly regulated at various cellular stages (Roundtree et al., 2017b; Zaccara et al., 2019). Methyltransferase is a multi-component protein primarily comprising methyltransferase-like 3 (METTL3), −14 (METTL14), and Wilms tumor 1-associated protein (WTAP). KIAA1429, RBM15, RBM15B, ZC3H13, and CBLL1. m^6^A demethylation is primarily achieved by two enzymes: fat mass and obesity-associated protein (FTO) (Jia et al., 2011) and ALKB homolog 5 (ALKBH5) (Zhang et al., 2017). Generally, m^6^A “readers” are divided into three classes: the YTH family proteins (YTHDF1/2/3, YTHDC1/2), m^6^A conversion proteins (hnRNPC, hnRNPG, and hnRNPA2B1), and RNA-binding proteins (IGF2BP 1/2/3 and hnRNPA2B1) (Zaccara et al., 2019). Additionally, eIF3 and ELAVL1 are reported as “readers.” Several other m^6^A RNA binding proteins have been identified, such as LRPPRC, FMR1, FMRP, and SND1 (Chen et al., 2011; Darnell et al., 2011; Arguello et al., 2017).

## 3 Association of m^6^A with the inflammatory responses in metabolic syndromes

Metabolic syndromes are primarily associated with inflammatory responses during their development. For example, Day and James proposed the second-strike theory of nonalcoholic fatty liver disease (NAFLD). The “first strike” triggers a metabolic derailment of the mitochondria, endoplasmic reticulum stress, and hepatocyte peroxisomes. Reactive oxygen species (ROS) and further lipid peroxidation, cytokine production, and lipid accumulation promote inflammation and fibrosis. In atherosclerosis pathogenesis, oxidized low-density lipoprotein (ox-LDL) is absorbed by endothelial macrophages to form foam cells, causing inflammatory lesions in the endothelium and leading to atherosclerosis. Diabetes mellitus pathogenesis also involves the interleukin (IL)-1β inflammatory factor that damages islet cells and causes insufficient insulin secretion. Therefore, we focus on the impact of m^6^A methylation on inflammatory-related responses in metabolic syndromes.

### 3.1 Role of m^6^A on lipogenesis

The immune response induced by chronic inflammation is involved in T2DM, NAFLD, and atherosclerosis progression (Yvan-Charvet et al., 2019; Qin et al., 2021). Guo’s study on m^6^A methylation transcriptional profiles between the normal state and acute inflammation induced by lipopolysaccharide (LPS) in chicken liver potentially identified how m^6^A regulates acute inflammation and abnormal lipid metabolism (Guo et al., 2022). Similarly, METTL3 influences the uptake of long-chain fatty acids in the intestinal epithelium by affecting the inflammatory response induced by tumor necrosis factor receptor-associated factor 6 (Zong et al., 2019).

Macrophage-related research has also identified the regulatory functions of m^6^A modifications in inflammation. For example, lipid deposition causes atherosclerosis and NAFLD, which can be attributed to the metabolic reprogramming induced by macrophages due to the inflammatory response. Macrophages absorb and metabolize excess ox-LDL, producing esterified cholesterol in the cytoplasm and producing foam cells (Yvan-Charvet et al., 2019). Macrophage scavenger receptor 1 (MSR1) and cluster determinant 36 (CD36) are highly expressed on macrophage surfaces. These are the primary receptors for the binding, uptake, and removal of cholesterol. Ox-LDL induces dead box protein 5 (DDX5) expression, promoting MSR1 expression in macrophages and inhibiting the methyltransferase METTL3 activity in MSR1 and CD36 knockout mice. Subsequently, MSR1 mRNA stability is enhanced, and lipid uptake is promoted (Kunjathoor et al., 2002; Yvan-Charvet et al., 2019).

**FIGURE 2:**
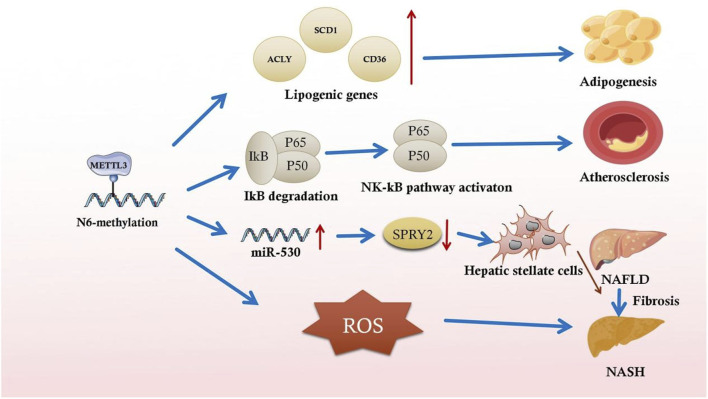
Partial illustration of the association between m^6^A methylation and inflammatory responses in metabolic syndromes.

In addition, lipogenesis-related gene mRNAs undergo m^6^A methylation. Activating the transcription factor sterol regulatory element binding protein-1C and lipid-responsive nuclear receptor liver X receptor through m^6^A in NAFLD animal models results in lipid accumulation in the liver (Salisbury et al., 2021). This lipid accumulation exacerbates NAFLD progression to nonalcoholic steatohepatitis (NASH) (Salisbury et al., 2021). m^6^A modification stabilizes ATP citrate lyase and stearoyl-CoA desaturase one mRNA, increasing protein expression in a NAFLD DM2 mouse model (Yang et al., 2022) (Figure 2). After that, these mRNAs exacerbate fat acid synthesis and lipid accumulation, leading to excessive compensatory cell proliferation in the liver and NAFLD and hepatocellular cancer progression (Yang et al., 2022). The m^6^A reader YTHDC2 is also critical for regulating hepatic adipogenesis and triglyceride homeostasis in NAFLD and NASH, which may provide a potential target to treat obesity-associated NAFLD (Zhou et al., 2021a). It has also been shown that Mettl3-IGFBP2-mediated changes in m^6^A levels increase HDAC1 mRNA stability to affect FGF21 expression, leading to liver injury and insulin resistance caused by hepatic steatosis and ultimately the development of metabolic syndrome (Chen et al., 2023).

These studies demonstrate the critical role of the m^6^A-mediated inflammatory response in lipogenesis, indicating the potential effects (28) of m^6^A modification on abnormal lipid metabolism.

### 3.2 Role of m^6^A on fibrosis

Recent studies have indicated the underlying function of m^6^A-mediated inflammation in fibrosis. Serum LPS concentrations are increased in high-fat diet-induced rats with NASH, and global m^6^A methylation upregulation is closely associated with this increase. LPS-activated Kupffer cells aggravate NAFLD progression to NASH and exacerbate fibrosis in chronically inflamed liver (Yang et al., 2022). In the early stages of liver fibrosis, the local inflammatory response activates acid-sensing ion channel 1a (ASIC1a), leading to stellate cell activation and proliferation, aggravating liver fibrosis (Feng et al., 2021). By altering the specific RNA m^6^A methylation mechanism, ASIC1a regulates miR-350 through METTL3-dependent m^6^A modification. By increasing miR-350 levels, ASIC1a suppresses the target gene sprouty receptor tyrosine kinase signal antagonist two expression, further mediating PI3K/AKT and ERK pathway activation and facilitating hepatic stellate cell activation (Figure 2) (Zhu et al., 2020).

Additionally, the immune response and apoptosis are critical factors in liver fibrosis regression. m^6^A methylation regulates oxidative stress and cytoplasmic metabolism in liver fibrosis mouse models related to hepatocyte immune responses and apoptosis, respectively (Cui et al., 2020). Increased m^6^A in RNA was also observed in the failing hearts of humans, pigs, and mice. In failing mammalian hearts and hypoxic cardiomyocytes, increased m^6^A levels reduce FTO levels. FTO overexpression decreases cardiac fibrosis in mice with myocardial infarction and demethylates the contractile transcript SERCA2a (Mathiyalagan et al., 2019). Cardiac fibroblast activation parallels high METTL3 expression, whereas silencing METTL3 substantially attenuates cardiac fibrosis and reduces collagen-related gene expression (Li et al., 2021a). Similarly, METTL3 deletion restricts the conversion of lung fibroblasts to myofibroblasts *in vitro* and *in vivo*; however, the regulatory mechanism was not related to m^6^A modification.

Therefore, further research on m^6^A modification-related inflammation and fibrosis is required. A better understanding of this topic will provide new insights into fibrotic disease treatment.

### 3.3 Role of m^6^A on metabolic-related cell signaling pathways

m^6^A modification influences the inflammatory state at the cellular level through signaling pathways in an established macrophage polarization system using RAW264.7 macrophages and bone-marrow-derived macrophages (BMDMs) (Liu et al., 2019a; Gu et al., 2020). Macrophages are the primary cell types involved in inflammation. METTL3 knockdown suppresses inflammatory cytokine production and expression of various genes, primarily by altering the phosphorylation levels of relevant signaling pathways in BMDMs (Liu et al., 2019b). For example, in atherosclerosis, myeloid differentiation factor 88 (My88) expression is induced by LPS METTL14 upregulates the My88 phosphorylation level and exacerbates vascular endothelial inflammation, suggesting that METTL14-related m^6^A is involved in atherosclerotic inflammation (Zheng et al., 2022). METTL3 potentially exerts a pro-inflammatory effect through m^6^A-derived macrophage polarization to the pro-inflammatory M1 type (Liu et al., 2019b; Wang et al., 2019). Nevertheless, in LPS-induced macrophages, METTL3 upregulation substantially attenuated NF-κB signaling pathway-dependent inflammatory responses in METTL3 knockout mice (Wang et al., 2019; Yu et al., 2019). Additionally, inhibiting demethylases affected the phosphorylation of essential proteins, including IKK α/β, IκBα, and p65, in the NF-κB signaling pathway. Demethylase inhibition also decreases STAT1 and PPAR-γ mRNA stability and thus hinders macrophage polarization (Gu et al., 2020). In contrast, METTL3 increases critical transcription factor expression, such as STAT1, to initiate pro-inflammatory macrophage polarization and induce M1 macrophages in various inflammatory diseases (Liu et al., 2019b).

Regarding metabolic disorders, m^6^A modification-regulated inflammation-related signaling pathways have been elucidated. METTL3 was highly expressed in type 1 and 2 diabetic nephropathy. Increased m^6^A levels further enhance TIMP2 stability through an IGF2BP2-dependent mechanism, leading to cellular inflammation and apoptosis via Notch3/4 pathway activation (Jiang et al., 2022). Furthermore, m^6^A activates macrophages and reprograms cellular metabolism during NAFLD progression. METTL3-mediated m^6^A in Kupffer cells downregulates DNA damage-inducible transcript 4, leading to NF-κB pathway activation and upregulation of inflammation in the liver. This inflammation ultimately increases lipogenesis and obesity and facilitates the inflammatory progression of NAFLD (Qin et al., 2021).

In summary, the m^6^A-mediated inflammatory response to cell signaling pathways is critical in metabolic syndromes. However, the exact effect of m^6^A on inflammation in various metabolic syndromes remains unclear. Therefore, further studies are required to better elucidate the m^6^A-related pathogenesis of metabolic syndromes.

## 4 m^6^A regulates metabolic syndromes through PCD

PCD, in addition to typical cell death modalities such as autophagy, ferroptosis, necrosis, and thermoproteolysis, is a programmed mechanism that eliminates abnormal cells to maintain the balance of the internal environment. Autophagy is an evolutionarily conserved degradation pathway that is primarily free of unnecessary and senescent organelles and proteins and is tightly regulated by autophagy-associated proteins and transcription factors. Autophagic dysregulation is associated with many diseases, such as neurodegenerative syndromes, cardiovascular diseases, and cancer, owing to its several cytoplasmic targets. Furthermore, autophagy is a normal cell function in metabolic syndrome development and progression. Therefore, we focus on the impact of m^6^A methylation in PCD in metabolic syndromes.

### 4.1 Association of m^6^A methylation and autophagy

**FIGURE 3:**
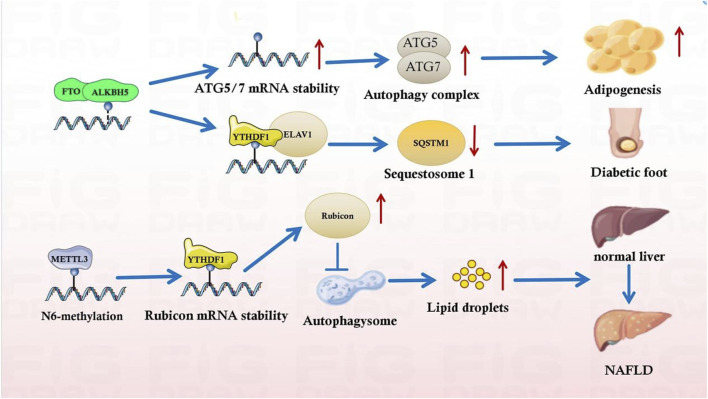
m^6^A influences the metabolic syndrome progression by regulating autophagy.

Several autophagy-related genes are involved in the mRNA processing of m^6^A modifications. Post-transcriptional ATG1 and ATG5/ATG7 regulation can be altered by m^6^A modification, thereby inhibiting autophagy (Figure 3) (Wang et al., 2020). Furthermore, many studies have demonstrated the role of m^6^A modification in autophagic machinery. Recent data suggest that m^6^A is critical for regulating autophagy (Li et al., 2020). Some m^6^A modifications inhibit autophagy directly (Song et al., 2019), which may also affect autophagosome formation, thereby dysregulating autophagy in *Fto* ^flox/flox^ and *Fabp*4-Cre transgenic mice (Wang et al., 2020). m^6^A modifications may also promote autophagy initiation (Feng et al., 2021). Moreover, the effect of m^6^A modification on autophagy is related to the disease. As m^6^A modification and autophagy play critical roles in regulating health status, a better understanding of this topic is crucial to developing therapeutic strategies.

m^6^A harmonizes metabolic syndrome progression by affecting autophagy. METTL3 inhibition promoted hepatic autophagy and lipid droplet (LD) clearance. METTL3 directly mediate m^6^A modification of Rubicon mRNA, thereby promoting Rubicon mRNA stability. Consequently, Rubicon reduces autophagosome-lysosome fusion, further impairing LDs clearance and leading to liver lipid deposition, hepatic steatosis, and progression to NASH (Peng et al., 2022). Similarly, in a glioblastoma (GBM) stem cells (GSCs) related work, platelet-derived growth factor (PDGF) ligand stimulate early growth response 1 (EGR1) transcription to induce METTL3 to promote GSC proliferation and self-renewal. Targeting the PDGF-METTL3 axis inhibits mitophagy by regulating m^6^A modification of optineurin (OPTN) (Lv et al., 2022). Also, the adriamycin (ADR) mechanism in diabetic nephropathy treatment is related to m^6^A-regulated autophagy in ADR nephropathy in adult male C57BL/6 J mice (Lu et al., 2021). METTL14 downregulation reduces SIRT1 degradation in the presence of ADR. SIRT1 upregulates autophagy in podocytes; this upregulation initiates a stress-protective mechanism in podocytes, effectively alleviating podocyte damage and delaying diabetic nephropathy progression in METTL14 knockout mice (Lu et al., 2021). m^6^A-binding proteins also perform separate functions in regulating autophagy. In a high-glucose-induced diabetic retinopathy model, circFA T1 overexpression upregulated autophagy in retinal pigment epithelium (RPE) cells. By binding to the m^6^A reader YTHDF2, circFA T1 alleviates diabetic retinopathy (Huang et al., 2022). Notably, a similar protective mechanism was observed in pancreatic islet β-cells. Hypoxia-inducible factor 1-alpha upregulated ATG5, ATG2A, and ATG14 in a YTHDF1-dependent manner, triggering protective autophagy and ameliorating hypoxia-induced cytotoxicity (Fang et al., 2022). Therefore, m^6^A aggravates metabolic syndromes by slowing down autophagy and cell damage in metabolic-related diseases by initiating cytoprotective autophagy mechanisms.

m^6^A modification harmonizes metabolic syndromes through the autophagic pathway. A recent study on adipocytes reported that FTO deletion decreased ATG5 and ATG7 expression, the mechanism of which is related to the m^6^A modification downregulation (Wang et al., 2020). ATG7 undergoes ATG12/ATG5 covalent binding to a ubiquitin-like mechanism. The ATG12-ATG5 homodimer attaches to ATG16L and facilitates the autophagosome extension in BALB/c nude mice and non-small cell lung cancer cell lines PC9 and HCC827 (Liu et al., 2020). FTO deprivation reduces ATG12-ATG5 covalent binding, impedes ternary complex development, and impairs autophagy activation (Wu et al., 2020). Autophagy reduction mediated by FTO and ATG gene expression suggests an association between m^6^A modification and autophagy. Furthermore, SQSTM1 is a multifunctional protein vital to autophagy (Moscat and Diaz-Meco, 2009). As a critical signaling center, SQSTM1 activates the mechanistic target of rapamycin kinase complex 1, kelch-like ECH-associated protein 1-nuclear factor, and the erythroid 2-like two pathway in addition to selective autophagy (Katsuragi et al., 2015). YTHDC1 cooperatively regulates SQSTM1 expression in keratinocytes. In patients with diabetes, YTHDC1 downregulation induces decreased SQSTM1 expression through accelerated SQSTM1 nuclear mRNA decay, leading to disturbed autophagic flux and keratinocyte migration, thus delaying wound healing (Figure 3) (Moscat and Diaz-Meco, 2009; Katsuragi et al., 2015).

### 4.2 m^6^A regulates metabolic syndromes through apoptosis

m^6^A levels were significantly reduced in the total RNA of mouse islet cells treated with hydrogen peroxide (H_2_O_2_) to mimic the ROS environment. H_2_O_2_ treatment significantly reduced METTL3 and METTL14 expression, whereas METTL3 interference enhanced cleaved caspase-3 protein and the pro-apoptotic protein Bim expression. This finding suggests that the ROS environment simulated by H_2_O_2_ may reduce m^6^A methylation in pancreatic cells, resulting in apoptosis. Deleting islet cell-specific METTL3 (Mettl3^flox/flox^, Rip-Cre) is related to cell death and dysfunction, indicating that METTL3 is vital for β-cell survival (Li et al., 2021b). Therefore, METTL3 may be more critical than METTL14 in regulating pancreatic β-cell function. Furthermore, the blood glucose level significantly increased in METTL3 knockout mice (Mettl3^flox/flox^, Rip-Cre) compared to METTL14 knockout mice (Liu et al., 2019c; Li et al., 2021b). Similarly, the m^6^A-binding protein YTHDF1 inhibits β-cell apoptosis, suggesting that m^6^A-related proteins are critical in β-cell death (Fang et al., 2022). Furthermore, myocardial lipid accumulation promotes myocardial apoptosis (Lee et al., 2013). Cleaved caspase-3 protein expression in the myocardium of obese rats increases, indicating increased apoptosis (Sun and Zhang, 2021). FTO mRNA and protein expression are upregulated in obese rats and are associated with disturbed lipid metabolism in LO2 cells (Guo et al., 2013). Furthermore, restricting high-fat foods reduced cleaved caspase-3 protein expression, inhibiting high-fat diet-induced apoptosis in the hearts of obese rats. Similarly, cardiac lipid deposition significantly increases cardiac apoptotic cell death, and dietary intervention reverses these effects. It has been shown that YTHDF2-mediated SIRT3 increases vascular endothelial cell apoptosis and ultimately contributes to the development of diabetic atherosclerosis (Zhang et al., 2023). High glucose-treatment regulates PINK1 expression in response to Mettl3-YTHDF2 in renal tubular epithelial cells, which in turn causes renal tubular epithelial cell apoptosis. This study may reveal that m6A-mediated apoptosis contributes to the pathogenesis of diabetic nephropathy (Wang et al., 2023a).

## 5 m^6^A regulated metabolic syndrome through pyroptosis

Inflammatory vesicle-mediated pyroptosis leads to cell death characterized by activating multiple caspases, including caspase-1, in an immortalized mouse podocyte cell-5 line (Liu et al., 2021). m^6^A is essential in regulating intracellular pyroptosis based on the human RPE cell line ARPE-19 (Zha et al., 2020). Nod-like receptor protein 3 (NLRP3) is a crucial inflammatory vesicle component that causes pyroptosis and increases pro-inflammatory cytokine levels (Yu et al., 2019). High sugar levels induce the increased expression of heat degradation-related proteins (caspase-1, gasdermin D, NLRP3, IL-1β, and IL-18), leading to pyroptosis (Mulay, 2019). These proteins are less expressed when METTL3 is overexpressed and cellular damage is reduced. In contrast, the damage is exacerbated in human peripheral blood mononuclear cell-derived macrophages when METTL3 is knocked down (Guo et al., 2020). A previous study investigated how interferon regulatory factor-1 (IRF-1) facilitates macrophage scorching among patients with acute coronary syndrome, and elevated m^6^A and METTL3 levels were observed in macrophages (Kunjathoor et al., 2002). IRF-1 overexpression increases m^6^A and METTL3 levels and promotes acute coronary syndrome (Liu et al., 2019c). According to a study on disc degeneration, METTL14 specifically induces the NLRP3 mRNA m^6^A modification and increases NLRP3 protein expression in the human osteosarcoma cell line U2OS (Yuan et al., 2021). Therefore, we hypothesized that METTL3/14 plays a joint role in pyroptosis death by regulating NLRP3, thus influencing metabolic syndrome development.

Ferroptosis, a new type of programmed cell death, involves iron-dependent lipid peroxidation and glutathione peroxidase 4, and mitochondrial membrane loss. m6A is vital in ferroptosis as a novel post-transcriptional regulatory mechanism (Shen et al., 2021). YTHDF1 enhanced the BECN1 stability by binding to the BECN1 m6A site and activating hepatic autophagic vesicle formation in primary hepatic stellate cells from ICR mice. Autophagic vesicles trigger ferroptosis in stellate cells and attenuate liver inflammation (Shen et al., 2021). METTL3/ASK1-p38 pathway-induced ferroptosis might be one of the leading causes of diabetic osteoporosis induced by high sugar and a high-fat diet (Lin et al., 2022). m6A modification induces diabetic erectile dysfunction by regulating ferroptosis in penile cells in a diabetes mellitus with erectile dysfunction model in SD rats (Wang et al., 2023b). ALKBH5-mediated m6 A demethylation was reported to lead to the posttranscriptional inhibition of NFE2L2/NRF2, which is crucial for the regulation of antioxidant molecules in cells. Knocking down ALKBH5 subsequently increased the expression of NFE2L2/NRF2 and increased the resistance of HPSCC cells to ferroptosis (Ye et al., 2022). In a diabetic cataract (DC) research, RBM15, WTAP, ALKBH5, FTO, and YTHDF1-were upregulated in DC samples, the mechanism of which is related with the ferroptosis pathway (Cai et al., 2023).

Glucolipotoxicity is toxicity caused by elevated glucose and fatty acid levels, frequently occurring during diabetes development due to hyperglycemia and hyperlipidemia. Autophagy is activated as an adaptive response and provides a possible protective mechanism for β-cells to eliminate damaged mitochondria, unwanted proteins, or both to avoid dysfunction and apoptosis (Meijer and Codogno, 2007). However, glucolipotoxicity induces excessive autophagy in β-cells, leading to cell death. RNA demethylation of critical autophagy pathway genes is an essential factor of β-cells dysfunction and the pathophysiology of diabetes. FTO-m6A demethylation is responsible for NR3C1-stimulated ATG gene expression under glucolipotoxic conditions, triggering excessive autophagy and β-cell death (Wu et al., 2023).

m^6^A methylation exacerbates metabolic syndrome progression by affecting programmed cell death. In diabetes and its complications, m^6^A-mediated apoptosis, ferroptosis, and excessive autophagy-induced-cell and osteoblast damage are particularly important. Similarly, markers related to programmed cell death indicate that m^6^A is a potential emerging target for detecting and treating metabolic syndromes.

To summarize, we included Table 1 to present the m^6^A-related proteins that play essential roles in modulating metabolic syndrome development. Table 1 also summarizes the target genes related to these proteins. These essential proteins will likely become vital targets for m^6^A in clinical applications.

**TABLE 1:** m^6^A-related proteins in metabolic disease.

m^6^A-related proteins	Target gene	m^6^A regulators level	Metabolic disease
METTL3	ACLY/SCD1	Down	NAFLD Yang et al. (2022)
METTL3	TRAF6	Down	Uptake long-chain FAs Zong et al. (2019)
METTL3	MSR1	Down	Uptake of ox-LDL by foam cells Yvan-charvet et al. (2019)
METTL3	SPRY2	Up	Liver Fibrosis Zhu et al. (2020)
FTO	SERCA2a	Down	Myocardium Fibrosis Mathiyalagan et al. (2019)
METTL3	TIMP2	Up	Diabetic nephropathy Jiang et al. (2022)
METTL3	DDIT4	Down	NAFLD Yu et al. (2019)
METTL3	Rubicon	Up	NAFLD Lv et al. (2022)
METTL14	Sirt1	Up	Diabetic nephropathy Lu et al. (2021)
YTHDF2	CircFAT1	Down	Diabetic retinopathy Huang et al. (2022)
YTHDC1	SQSTM1	Down	Diabetic foot Katsuragi et al. (2015)
METTL3/14	—	Up	β-cell death
FTO	Caspase3	Up	—
YTHDF1	BECN1	Down	Liver Fibrosis Shen et al. (2021)
METTL3	ASK1	Up	Diabetic osteoporosis Lin et al. (2022)
FTO	NR3C1	Up	β-cell death Wu et al. (2023)

## 6 Discussion

RNA methylation is one of the most common post-transcriptional modifications. m^6^A regulates transcription, translocation, splicing, and translation. m^6^A modifications are commonly detected in biological processes using RNA-seq and methylation RIP-seq (or miCLIP-seq) to explore the role of m^6^A modifications in metabolic syndromes. m^6^A modification is reversible using m^6^A regulators (methylesterases, demethylases, and m^6^A-RNA binding proteins).

Comparative observations across disease conditions have revealed that the m^6^A modification levels and their associated regulators vary in T2DM, NAFLD, and atherosclerosis. However, further studies are needed to identify the exact association between m^6^A modification and the pathogenesis of metabolic syndrome-mediated regulatory factors. In addition, m^6^A RNA modification is tissue-specific, and whether it is specific to the disease stage remains unclear. Currently, treating metabolic syndromes involves exercise and controlling a high-calorie diet. Although weight-loss surgery effectively slows the progression of metabolic syndromes, it is not widely used because it harms patients. Studying the m^6^A levels in peripheral blood samples from patients with metabolic syndromes can help identify early biomarkers. Although only confirmed in cellular experiments and animal tests in many studies, these m^6^A-related markers can become therapeutic alternatives to weight loss surgery in the future. This potential therapeutic alternative can treat patients with metabolic syndromes without the adverse events of surgery.

We summarized the Abbreviation list in Table 2 to better understand the present review. m^6^A-related proteins, especially METTL3, are critical in regulating m^6^A in inflammation, autophagy, and cell death. We believe that targeting m^6^A-related proteins may be ideal for developing metabolic syndrome treatments (Yankova et al., 2021). For example, in cellular and animal studies, STM2457, a small molecule active inhibitor of METTL3, inhibits acute leukemia development caused by elevated m6A levels. Drugs such as these inhibitors may shed new light on treating metabolic syndromes at the epigenetic level. In recent years, some m^6^A-related clinical trials have been progressively reported (Luo et al., 2020; Cai et al., 2021; Yang et al., 2021). We concluded the information in Table 3. Such as, it has been shown that SNP rs34269950 in the RRACH genome, located in the 3′UTR of RUNX1T1, is significantly regulated by FTO in rural populations in the Caucasus region of Australia, providing important clinical evidence for the association of m6A with metabolic syndrome (Zhou et al., 2021b).

**TABLE 2:** Abbreviations.

Full spelling	Abbreviations
N6-methyladenosine	m^6^A
messenger RNA	mRNA
noncoding RNA	ncRNA
type 2 diabetes mellitus	T2DM
nonalcoholic fatty liver disease	NAFLD
endoplasmic reticulum stress	ER
Reactive oxygen species	ROS
Programmed cell death	PCD
autophagy-associated proteins	ATG
methyltransferase-like 3	METTL3
methyltransferase-like 14	METTL14
Wilms tumor 1-associated protein	WTAP
S-adenosylmethionine	SAM
Fat Mass and Obesity-Associated protein	FTO
ALKB homolog 5	ALKBH5
lipopolysaccharide	LPS
Macrophage scavenger receptor 1	MSR1
dead box protein 5	DDX5
liver X receptor	LXR
nonalcoholic steatohepatitis	NASH
hepatocellular cancer	HCC
myocardial infarction	MI
myeloid differentiation factor 88	My88
DNA damage-inducible transcript 4	DDIT4
lipid droplet	LD
Adriamycin	ADR
mechanistic target of rapamycin kinase	mTOR
mediates activations of mechanistic target of rapamycin kinase complex 1	mTORC1
kelch-like ECH-associated protein 1	KEAP1
nuclear factor, and erythroid 2 like 2	NFE2L2
cluster determinant 36	CD36
Sterol regulatory element binding protein-1C	SREBP1C
ATP citrate lyase	ACLY
stearoyl-CoA desaturase1	SCD1
sprouty receptor tyrosine kinase signal antagonist 2	SPRY2
tumor necrosis factor receptor-associated factor 6	TRAF6
retinal pigment epithelium	RPE
YTH domain family	YTHDF
Insulin-like Growth Factor 2 mRNA-binding Protein	IGF2BPs
heterogeneous nuclear ribonucleoprotein	hnRNPs

**TABLE 3:** Reports of clinical trials.

Diseases	Target	Results	References
Obesity	FTO/RUNX1T1 rs34269950	RUNX1T1 rs34269950 located in the m^6^A motif may influence abdominal obesity	Zhou et al. (2021b)
Rheumatoid Arthritis (RA)	ALKBH5, FTO, and YTHDF2	Demethylase ALKBH5 and FTO were associated with RA	Luo et al. (2020)
Gastric cancer (GC)	FTO/m^6^A/MYC	FTO/m^6^A/MYC axis may participate in the regulation of GC	Yang et al. (2021)
Hepatocellular carcinoma (HCC)	small nuclear ribonucleoprotein polypeptide C (SNRPC)	SNRPC was mainly related to protein metabolism and the immune process and linked to a worse prognosis in patients with HCC	Cai et al. (2021)

In conclusion, m^6^A RNA modification regulates many factors involved in the pathogenesis of metabolic syndromes and may play a vital role in metabolic syndrome development and progression. However, many studies are still needed to answer multiple outstanding questions, such as whether specific inhibitors possess clinical functions in metabolic disorders, how to achieve tissue-specific drug delivery, and whether side effects exist during m^6^A RNA modification regulation. Clarifying these research questions will provide a new frontier in epigenetic modification and metabolic syndrome research.

## 7 Conclusion and future direction

In the future, the detection of m^6^A and its regulatory molecules will also be developed to be more convenient, with a wider range of samples and higher sensitivity, and closer and closer to bedside detection. As basic research on m^6^A and metabolic sydromes intensifies, more and more basic findings will be confirmed in clinical trials. More convenient detection Methods will facilitate these clinical trials, and clinical trials will provide evidence for drug development targeting m^6^A regulators. With the maturation of screening technology and the development of artificial intelligence (AI), more and more drugs targeting m^6^A regulatory molecules will be discovered. The study of Chinese medicinal decoction for m^6^A regulatory factors will also become a direction for future drug research on m^6^A-related metabolic sydromes.
